# Defining a common set of indicators to monitor road accidents in the European Union

**DOI:** 10.1186/1471-2458-6-183

**Published:** 2006-07-11

**Authors:** Sara Farchi, Nunzio Molino, Paolo Giorgi Rossi, Piero Borgia, Michael Krzyzanowski, Dafina Dalbokova, Rokho Kim, the European Road Accident Indicator Working Group

**Affiliations:** 1 Department of Prevention, Agency for Public Health of the Lazio Region, Rome, Italy; 2 WHO European Centre for Environment and Health, Bonn, Germany

## Abstract

**Background:**

currently road accidents are mostly monitored through mortality and injury rates. This paper reports the methodology and the results of a project set forth by the European Union (EU) and coordinated by the WHO aimed at identifying and evaluating a core set of indicators to monitor the causal chain of road accident health effects. The project is part of the ECOEHIS (Development of Environment and Health Indicators for European Union Countries).

**Methods:**

a group of experts (WG), identified 14 indicators after a review of the information collected at the EU level, each of them representing a specific aspect of the DPSEEA (Driving, Pressure, State, Exposure, Effect, Action) model applied and adapted to the road accidents.

Each indicator was scored according to a list of 16 criteria chosen by the WG. Those found to have a high score were analysed to determine if they were compatible with EU legislation and then tested in the feasibility study.

**Results:**

11 of the 14 indicators found to be relevant and compatible with the criteria of selection were proposed for the feasibility study. Mortality, injury, road accident rate, age of vehicle fleet, and distance travelled are the indicators recommended for immediate implementation.

**Conclusion:**

after overcoming the limitations that emerged (absence of a common definition of death by road accident and injury severity, underestimation of injuries, differences in information quality) this core set of indicators will allow Member States to carry out effective internal/external comparisons over time.

## Background

### The burden of road traffic accidents in Europe

Traffic accidents cause about 120 000 deaths and 2.5 million injuries a year in Europe. It has been estimated that the total cost to society is higher than €160 billion a year, approximately 2% of the EU GNP [1].

Road traffic accidents are the most common cause of death among young people, especially males, and they are a leading cause of physical disability [2]. Reducing the number of traffic accidents and the resulting injuries and deaths is a priority throughout Europe [3]. It is particularly urgent in Central-Eastern European countries where improvements in traffic infrastructures have not kept up with the rapidly growing traffic density [4].

### The ECOEHIS (Development of Environment and Health Indicators for European Union Countries) project

ECOEHIS [5] is a project conducted by the WHO to develop methods and tools for the Environmental and Health Information System (EHIS). The project objective was to establish a core set of environmental health (EH) indicators for the European Union (EU) countries, covering the topics related to important areas of living conditions such as air pollution, noise, housing conditions, road accidents and water pollution [6]. Eleven member States (out of 15) participated in the project with a delegate from the Health and Environment Ministries.

The first phase of the project identified two indicators in the road traffic injury causal chain. The two indicators, mortality rate and injury rate, both described the health effects of road traffic accidents, while no indicator analysed driving forces and pressures influencing this public health relevant problem. The necessity of revising the core set of Environmental and Health indicators, with a new EC DG Sanco financial support, provided the opportunity to conduct a thorough analysis of the causal chain of road traffic accidents.

The objective of the sub-project is to establish a core set of environmental health indicators for EU countries. To achieve this, the project proposes, validates and tests the feasibility of collecting the indicators ;moreover one of the important aims of this project is to assure that the proposed set is consistent with the existing body of regulations and legislation at the EC level.

The indicators will contribute to the establishment of a "community monitoring system" in order to:

 Measure the road accidents phenomenon, its determinants and the trends throughout the community

 Facilitate the planning, monitoring and evaluation of the relevant community programs and actions

 Provide member states and other international organizations with appropriate information to make comparisons and support their policies.

### Objective of the paper

Aims of this paper are:

to present the final set of indicators on road traffic injuries to be included in the Environmental and Health Information System (EHIS) for Europe;

to describe the process that allowed a group of researchers, public health operators and decision makers to build an evidence-based set of indicators using a sound methodology.

## Methods

### The review

A review was performed of the existing Information Systems that collect data on road accidents in the Member States, and of indicators identified in the literature (table 1). The review consisted of

 a systematic review of the official information systems used for official statistics on road accidents; the first step of the strategy was a search of the single Member State Institutes collecting data for the EURO-CARE [7] project, then a search in the official web sites of the Ministries of Transport and Health and of the National Statistic Agencies; finally a formal contact to the data reporting offices of the competent Ministry of each Member State to check the information gathered;

 a non systematic review of the more relevant experiences of other systems collecting relevant information for the road accidents;

 a review of the relevant data collection at the national and the local levels.

### The Working Group (WG)

A group of experts was established representing individual technical disciplines, information scientists and data providers. All the institutions involved in the ECOEHIS project were asked to indicate a possible reference Agency/institution for their Country, all the Agencies indicated were asked to participate in the sub-project on a voluntary basis. Furthermore, experts involved in the ongoing related European projects (STAIRS, ECOSA, IRTAD) were asked to participate.

### The selection process of the set of indicators

According to the final scope of the indicators, the WG defined the evaluation criteria for the indicators. The ECOHEIS project adopted the DPSEEA [8] (Driving forces, Pressure, State, Exposure, Effect, Action) model as the framework to describe and interpret the causal chain of environmental causes and health effects. The WG decided to use the same model for road accidents as used in the main project for consistentency.

The indicators proposed were crosschecked with the evaluation criteria (reported in the first column of table 2) independently by each expert of the WG, the possible values were 'yes' or 'no'. The individual results were than summarized in a three-level qualitative score as follows: score 1, if all the WG members reported yes or missing; score 0, if all the WG members reported no or missing; score 0.5, in case of at least two discordant results. Indicators that were scored 0 for the public health relevance were excluded. A global score was calculated as the sum of the scores obtained by each criterion, which had a potential range of 0 to 16. Indicators that obtained a total score above 10 were proposed for implementation.

### Verification of compatibility of selected indicators with EU legislation

An extensive review of the European Community legislation was carried out relevant to the topics to verify their compatibility with the European Health Indicators. For each selected indicator, relation to the EC legislation, reporting obligations, planned modifications in legislation and the need for modification in indicators were examined. At the end indicators were classified into the following categories with EU legislation: not compatible, compatible, compulsory not harmonized, compulsory and harmonized.

### The feasibility study

The selected and compatible indicators were submitted to feasibility testing. National focal points, networks of experts set up at the national level by the Ministry of Health and the Ministry of Environment in all the 11 Member States partners in the ECOEHIS project, collected the information on the availability and quality of the data necessary for the indicators in their own countries using a structured questionnaire. Each indicator has been evaluated according to four criteria with a three-level score (0, poor; 1, fair; 2, good): availability, reliability, comparability, and policy relevance. In addition, overall readiness was assessed and classified as follows: a) immediately; b) by the end of 2004; c) by the end of 2005; d) after 2006. The results of the questionnaires were summarised, averaging the results from each country, and presented to the Member State delegates in a final meeting in Bonn [5]. After a plenary discussion of the results, the final set of indicators and a priority scheme for the implementation of indicators was established:

• Ready and recommended for immediate implementation (recommended as 'core' European Community Health Indicators).

• Ready, but not feasible for immediate implementation

• Desirable, though requiring further developmental work

## Results

### Review of existing information systems and indicators

Police reports were the main source of information in all of the member states. Reports are completed after accident on a public road that leads to personal injury, and then collected centrally by national statistics office that annually publicizes those statistics. There are only small differences in data collection methods among the member states, while the common reported characteristics are: the accident (location, time of occurrence, light conditions), the vehicle involved (category, type of road usage, power of engine) and person involved (age, sex, physical condition, use of safety devices, type of road user). In some Member States, official statistics are integrated with information gathered by census data, death certificates, coroner registration forms, and hospital inpatient surveillance systems.

Also surveys are used to gather information not routinely collected such as: distances travelled, person time spent on the road, driving conditions, use of safety devices, and other driving behaviours.

Other information systems are based on health data: trauma registries and emergency or hospital based surveillance systems.

Table 1 presents the indicators usually calculated on the basis of the information routinely collected by the national statistics offices

### Application of the DPSEEA model to the road traffic accident field (Figure 1 [see Additional file 1])

The DPSEEA framework has been used both as a way of selecting and to structure environmental health indicators. The model was adapted to the main topics related to road accidents for each model step, plus the two additional steps specific for road accidents: risk factors and event (crash). It recognizes that the link between exposures and health effects is determined by many different factors operating through a chain of events.

The WG decided to apply a bottom up strategy to contextualize the model to the road accident field.

####  Health effects

##### Mortality indicators

The mortality figures obtained from police reports may be affected by under-reporting, even though this phenomenon is less important than in morbidity figures. Integrating this data with the mortality registry, through record linkage, quantifies the under-reporting. Furthermore, it is important to determine the years of life lost that give a measure not only of the impact of mortality but also of the age composition, because road accidents all over Europe affect young people. Other indications were: to distinguish the deaths of tourists often reported in the numerator of mortality rate (whereas in the denominator only the resident population is counted), and to observe ten-year trends for children's injuries.

##### Injury

The morbidity obtained by police reports do not give information about severity and diagnosis, furthermore it is affected by substantial under-reporting. The use of Health System-based data combined with police reports may overcome this weakness.

Health Care Systems in different countries could affect the computation of indicators regarding injury; in some countries the emergency department is the most common solution especially for mild injuries, while in others, there are general practitioners or primary care centres [9,10].

The health effects should be measured also taking into account the **disability **related to road accidents; QALY (*Quality Adjusted Life Year*) or DALY (*Disability Adjusted Life Year*) should be considered. Finally, it will be revealing to take into account the **psychological **consequences of road accidents, with or without a physical injury.

####  The "event" (road traffic accident)

A key point of discussion was to establish the road accident position within the DPSEEA model.

We decided to extrapolate the road accident from the conceptual framework and to consider it as a "event" for the occurrence of health consequences. The WG adopted an accident definition as an event due to the rapid transformation of kinetic energy[11]. The number of accidents causing at least one injury must be gathered.

####  Risk factors

All exposures that modify the risk of health effects related to a road traffic accidents should be considered as risk factors. These factors act as primary, increasing or decreasing the probability of an accident, or secondary, increasing or reducing the damage after the accident. Some of them act at both levels simultaneously [12].

An initial list has been proposed of risk factors to be monitored: risky behaviours (speed, as an individual and collective risk factor, drunk driving, driving under the influence of legal and illegal drugs, newly licensed drivers, fatigue, driving at night; use of mobile phones while driving, use of walkman while driving, the presence of cyclists and pedestrians, auditory, visual and/or impairments of physical mobility, medical conditions, mental illness); vulnerable subjects (older road users, unsupervised children pedestrian); protective behaviours (use of seat belts, use of motorcycle helmets, use of bike helmets, child restraints, airbags and other passive protective devices); environment (driving on rural/urban roads; road infrastructures). We found 13 indicators covering some of the above-mentioned risk factors.

The proportion of deaths occurring in people exposed to some of these risk factors can be monitored by using data already collected for mortality statistics; the reliability of this information varies between countries.

####  Exposures

The peculiarity of the road accident causal chain is that there is no linear association between the effect and the exposure to the road traffic. In fact the behavioural and environmental risk factors presented before modulate the risk of travelling or being on the road.

Two main axis to measure exposure have been individuated:

○ The time of exposure to the road, information usually gathered by national surveys or census (person time spent on the road)

○ The distance travelled, usually calculated using the number of circulating vehicles and applying an estimate of the km travelled yearly and of persons transported on average (distance travelled). This indicator is usually computed by national statistics.

The proposed exposures can be used as denominators of all health effects, obtaining indicators of the actual risk for any activity on the road.

These two quantities can be stratified by different covariates, such as mode of road user, type and condition of the road.

####  State

State was represented by several topics regarding all conditions influencing the quantity of exposure to the road and the probability of an accident: degree of urbanization, relative location of homes, schools, shops, and work places, age and quality of the vehicle fleet, extension and quality of road network, and climate.

An indicator of the age of the vehicles is the *renewal rate of vehicle fleet*, which can be calculated for all countries. In some cases data is not timely or updated, for example the vehicle fleet resulting from the registry could include a significant proportion of vehicles no longer in circulation.

*The extent of the road network *can be obtained for all countries, although the definitions of the different types of roads are not homogenous.

####  Pressure

Cultural and social norms creating the interest in having a car, and driving were identified as pressure factors that derive from the "driving forces" listed below.

####  Driving forces

The principal driving forces identified were the factors creating the need to travel and to move:

○ Economic status of the country

○ Physical geography of the country

○ Distribution of the population and urbanization

○ Distribution of wealth

####  Actions

Actions include a very wide range of preventive interventions, policies, laws, structural changes, etc.

They can be aimed at reducing the health effects of accidents, reducing the prevalence of a risk factor in the population, reducing the person-time of exposure, or the number of accidents [13].

The WG, cohering to the objective of the EHI for EU project, agreed that the purpose of this set of indicators is to monitor the effect of actions on their target, more than to measure the presence of the action itself.

### The selection process

In order to select the final set of indicators, all the hypothetical indicators were screened according to the criteria defined. Two indicators were classified as not relevant ad one obtained an insufficient score (table 2). Eleven indicators were proposed for the feasibility study.

### Compatibility of indicators with EU legislation

As described in table 3, none of the eleven indicators identified for the feasibility study are incompatible with EU legislation. Some of them, besides being compatible, have great importance in prevention polices of Member states (% car exceeding speeds limits, use of safety devices), while other compulsory indicators are not presently harmonised between countries (road accident rate, injury) (table 3).

### The feasibility study

The results of feasibility study point out as good indicators are based on routinely collected data, they are available in most member states, reliable and scientifically-based, comparable over time and space, and useful for policy process. Among the indicators included in the final set 5 were considered ready and recommended for immediate implementation (table 4), according to the results of the questionnaires completed at the national focal points of the member states.

The indicator figures for the EU MS are reported in the Appendix (data sources: EUROSTAT [15] and CARE [7]).

## Discussion

### Limits of the study

This article is an attempt to build a bridge between decision makers and the scientific community. There is need for such communication; in fact, the scientific community tends to marginalize this kind of work or to apply strict or unrealistic criteria to evaluate the methodology, while policy makers have habitually paid little or no attention to the methodological background behind these kinds of projects. As a consequence the choices presented in this paper are not fully justified by rigorous criteria and were often influenced by necessary compromises: the choice of the model was due more to consistency with the rest of the ECOHEIS project than to a review of the literature; the criteria and the scoring system to evaluate the indicators was not validated; the results of the national focal points had to be decided in conjunction with the delegates by Health and Environment Ministries.

### The Model

The DPSEEA model represents a reliable framework to integrate the information gathered. It recognizes that the link between exposures and health effect are mediated by many different factors operating through a chain of events [17].

The DPSEEA model was developed from the effect of classical environmental exposures on health, i.e. air pollution and respiratory diseases. The application of this model to acute events such as injuries related to road accidents, is new and needs a sustained effort to conceptualise the cause-effect chain of accidents and injuries.

The WG decided to adapt the model to the complexity of the road accident phenomenon by first adding the "road accident" event ad the risk factors as additional steps in the causal scheme.

Up to date, few interventions, excepting those related to speed control and alcohol polices, were proven to be effective in reducing road accident health effects, and often effective interventions are linked to peculiar cultural and behavioural aspects and their results cannot be generalized, moreover some well structured but harmful interventions have been identified [13,18]. The WG then discussed the need to develop action indicators and decided not to propose them. The scope of the indicators was to monitor the changes introduced by policies, preventive programs, laws and other actions on road accidents more than to measure the presence of the action itself.

### The indicators

The WG stressed the intrinsic weakness of some of the indicators that are routinely calculated, pointing out the first cause of such a weakness is probably the source of information.

Police reports, the main source of information for all of the 15 MS, provide little information on health effects because their purpose is legal, not medical [19]. In particular, systematic misinformation about mild injuries underestimates the real burden of road accidents. The underestimate was calculated to be between 4 and 5 times lower than the incidence estimated through some health-based statistics.

The information gathered suffers, moreover, of conflicting definitions. In particular, data on deaths are limited by the absence of a clearly defined latency period and by the lack of distinction between road users; instead, data on injuries suffer from conflicting definitions of injury severity.

The review of existing data clearly showed on one hand the need to submit indicators already collected, in order to make them more reliable and realistic, on the other hand, it was clear that completing the current set by identifying other indicators was necessary.

At the end of this process 11 indicators were selected: each of them representing different steps in the causal chain. The renewal rate was related to the duration of road exposure and the probability of an accident (State). Time spent on the road (chosen in order to take into account the exposure of vulnerable categories, like pedestrians) and distance travelled cover the exposure link in the causal chain. The accident rate and the indicators of health effects of road traffic accident (injury rate and mortality rate) are present in almost all the MS, and therefore have been chosen to be part of the core set. Another very important effect indicator is the DALY, to take in account the enormous toll paid by the youngest. Use of safety devices, percentage of cars exceeding speed limits [20], and mortality due to drunk driving [21] are important indicators regarding behavioural aspects strongly associated with both the determinants and consequences of accidents.

All indicators selected were considered in the selection criteria to have the best performance and all were compatible with EU legislation

The set of road accident indicators proposed by the working WG for the feasibility study gave good results in terms of quality, availability, comparability and policy relevance. Out of 11 indicators proposed five were judged ready for implementation, and the others were considered relevant but needing further development.

Compared to the previous set, these indicators cover a wider range of the causal chain including upstream determinants. The new set of indicators cover important risk factors of road accidents such as speed, drunk driving and use of safety devices that are the targets of specific EU actions. Public health effects of road accidents on mortality and injury rates were further elaborated by computing PYLL (Potential Years of Life Lost) and DALY as separate indicators.

## Conclusion

The effort by the European Union should be considered an important step to better comprehend road accidents and to allow member states to carry out internal/external comparisons over time.

The clear definition of the selection criteria and the sharing of the process with the scientific and technical experts community is a necessary, even if not sufficient, condition to have feasible and useful indicators.

## Competing interests

The author(s) declare that they have no competing interests.

## Authors' contributions

SF, NM, and PGR collaborated on developing the methodology adopted to select the indicators and together wrote the paper. PB supervised the study and revised the manuscript. MK, DD, and RK all revised the manuscript and supervised the methodological aspects. The WG was involved in conceiving the methodology and revised the manuscript.

European Road Accident Indicator Working Group.: Camilloni Laura^1^, Chini Francesco^1^, Chironne Mireille^2^, Di Giorgio Maurizio^1^, Guasticchi G^1^, Mulder Saakje^3^, Pasquariello Carlo^4^, Quiroga Javier^5^, Romano Valeria^1^, Towner Elisabeth^6^, Vallet Gilles^2^, Ward Heather^7^.

^1^Agency for Public Health of the Lazio Region, Rome-Italy;

^2^INRETS-UMRETTE, Lyon-France ;

^3^Consumer Safety Institute, Amsterdam-The Netherlands;

^4^Ministry of internal affairs, Rome-Italy;

^5^Ayuntamento de Madrid, Dept. SAMUR (Area de Salud y Consumo), Madrid-Spain;

^6^Centre for Child and Adolescent Health, Hampton house, Cotham Hill, Bristol-UK,

^7^Centre for Transport Studies, University College London, London-UK.

## Pre-publication history

The pre-publication history for this paper can be accessed here:



## Supplementary Material

Additional File 1 DPSEEA model and selected indicators. Application of the DPSEEA model to the road traffic accident field. Click here for file

## Figures and Tables

**Figure 1:**
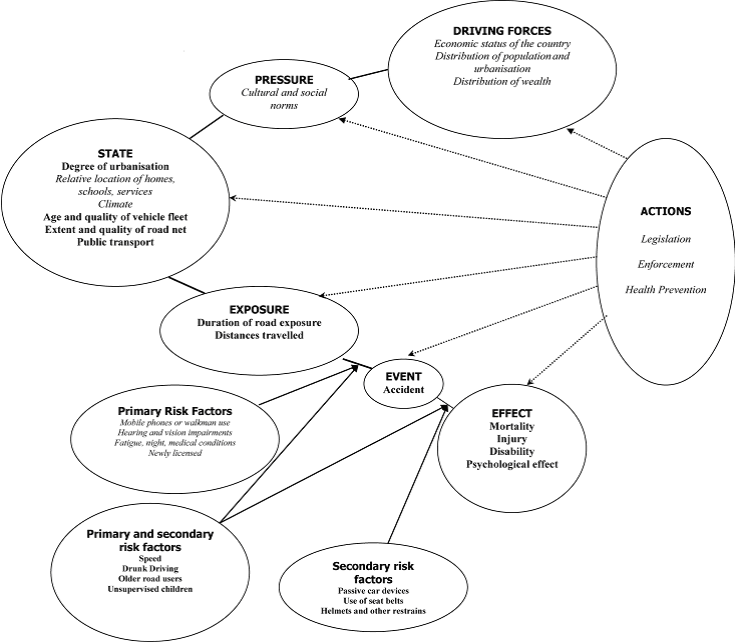
DPSEEA model and selected indicators. BOLD: topics relevant to the project aim. Italics: other topics not directly relevant to the project.

**Table 1:** Review of existing indicators on road accidents

**N.**	**Indicator**	**Computation**	**DPSEE model position**	**Data Sources**
**1**	Mortality rate due to traffic accident	**(M**_t_/P)*100,000 **M**_t _is the totalnumber of deaths due to traffic accidents **P **is the total population	**EFFECT**	**Data on deaths **are based on police records and death certificates
2	Pedestrian aged 10–14 dead/Population	**(M**_p__10–14_/P)*100,000 **M**_p_: is the total number of pedestrians dead because of traffic accidents **P **is the total population	**EFFECT**	**Data on deaths **are based on police records and death certificates
3	Death/motor vehicles	(Mt/V per year)*10,000 **M**_t _is the number of deaths due to traffic during a fixed period of time usually one year. **V **motor vehicle fleet in the country during the period of interest	**EFFECT**	**Data on deaths **are based on police records and death certificates **Data on vehicles **are provided by public motor vehicle registries
4	Death/km travelled	**(Mt/KM**_t_**) ** **M**_t _is the number of deaths due to traffic **Km**_t _total amount of km travelled for different categories of road users	**EFFECT**	**Data on deaths **are based on police records and death certificates **Data on km travelled **are based on surveys using questionnaires or odometer monitoring, or sales of fuel
5	Deaths/accident	**(M**_t_/A)*1,000 **M**_t _is the totalnumber of deaths due to traffic **A **is the total number of road traffic accidents	**EFFECT**	**Data on deaths **are based on police records and death certificates **Data on accidents **are based on police records
**6**	Death/km of road	**(M**_t_**/l**_r_)*1,000 **M**_t _is the totalnumber of deaths due to traffic **l**_r _is the length of the roads in Km	**EFFECT**	**Data on deaths **are based on police records and death certificates **Data on road length **are provided by ministries of transport and road directorates
**7**	Injury rate by traffic accident	**(I**_t_/P)*10000 **I**_t _is the total number of injured due to traffic accidents **P **is the total population	**EFFECT**	**Data on injuries **are based on police statistics, registration of medical care
8	Fatality rate	**(M**_t_**/M**_t_**+I**_t_**)** **M**_t _is the totalnumber of deaths due to traffic accidents **I**_t _is the total number of injured due to traffic accidents	**EFFECT**	**Data on deaths **are based on police records and death Certificates **Data on injuries **are based on police statistics, registration of medical care
9	Accident/vehicle	(A/V)*10000 **A **is the total number of road traffic accidents **V **is the total amount of vehicle	**EXPOSURE**	**Data on accidents **are based on police records **Data on vehicles **are provided by public motor vehicle registries
10	Vehicle fleet or (Motoritation index)	(V/P)*1000 **V **is the total number of vehicles **P **is the total population	**PRESSURE/STATE**	**Data on vehicles **are provided by public motor vehicle registries

**Table 2:** Results of the compatibility of indicators with the criteria selection

	**Mortality rate**	**Years of life lost**	**Injury rate**	**Distance travelled**	**Time spent on the road**	**Accident rate**	**Disability**	**Age of vehicle fleet**	**Extent of road net**	**Speed**	**Driving at night**
Definition	Mortality rate due to road accidents, by age and mode of road use	PYLL* attributable to road accidents	injury rate due to road accidents	Number of passenger Km travelled per year by mode of road use	Person time spent on the road by mode of road use	° of accidents involving injured people/pop or vehicles	DALY° lost attributable to road accidents	% of vehicle fleet renewal in a year	Km of roads by type of road	% of vehicles exceeding limits	° of cases 22–05 h/estimate of traffic at night

A clear and commonly accepted definition	y^†^	y	n^†^	y	n	y	y/n	y	y	y	y/n
Association with other Public Health indicators	y	y	y	y	y	y	y	n	n	n	n
Relevance	y	y	y	y	y	y	y	y/n	n	y	y
Power of discernment (ability to detect small changes in the phenomenon)	y	y	y	y	y	y	y	y/n	n	y	y/n
Sensitivity (depending on the source: % of detected cases on total existing cases)	y	y	n	y/n	y	y	y	y	y	y	y
Comparability in time	y	y	y	y	y/n	y	y/n	y	y	y/n	y/n
Comparability between countries	y	y	n	y/n	y	n	y/n	y	y	n	y/n
Timeliness (time elapsed from the event to the publication of the indicator)	y	y	y	y	y/n	y	n	y	y	y/n	y
Availability of information	y	y	y	y	y/n	y	y/n	y	y	y/n	y
Stability (how much is influenced by other factors, not regarding road accident field?)	y	y	y	y	y/n	y	y	y	y	y	y/n
Continuity (how long are the historical series for the indicator available?)	y	y	y	y	n	y	n	y	y	y/n	y/n
Cost effectiveness	y	y	y	y	y/n	y	y/n	y	y	y/n	y
Theoretical validity (how well the indicator represents the subject of interest.	y	y	y	y	y	y	y	y	y	y	y
Reliability (depending on the source: how good and valid is the figure given by the indicator)	y	y	n	y/n	y	y	y/n	y	y	y	n
Interpretability	y	y	y	y	y	y	y	y	y	y/n	y/n
Coverage (is the indicator available for all countries?)	y	y	y	y	n	y	y/n	y	y	y/n	y/n
Final score	16	16	12	14.5	10.5	15	10.5	14	n.r. §	10.5	10

**CRITERIA**	**Drunk driving**	**Driving rural urban**	**Children**	**Young drivers**	**Young drivers**	**Older road users**	**Use of seat belts**	**Use of helmets motorcycle**	**Use of helmets bike**	**Child restraints **	

Definition	° of drunk drivers inv in accident/pop	° cases in rural urban roads	° of cases 0–14/pop 0–14	° of cases 14–24 moped/pop 14–24	° of cases 17–24 car/pop 18–24	° of cases >70/pop >70	%of seat belt use in pop	%of helmet use in pop	%of helmet use in pop	%of restraint use in pop	

A clear and commonly accepted definition	y	y	y	y	y	y	y	y	y	y	
Association with other Public Health indicators	y	n	y	y/n	y/n	y	y	y	y/n	y	
Relevance	y	y	y	y	y	y	y	y	n	y	
Power of discernment (ability to detect small changes in the phenomenon)	y/n	y/n	y	y	y	y	y/n	y/n	y	y	
Sensitivity (depending on the source: % of detected cases on total existing cases)	y/n	y	y	y	y	y	y/n	y/n	y	y	
Comparability in time	n	y	y	y/n	y	y	y/n	y/n	n	y/n	
Comparability between countries	n	y/n	y	y	y	y	y/n	y/n	y/n	y/n	
Timeliness (time elapsed from the event to the publication of the indicator)	y	y	y	y	y	y	y	y	y	y	
Availability of information	y	y	y	y	y	y	y/n	y/n	y/n	y/n	
Stability (how much is influenced by other factors, not regarding road accident field?)	y	y/n	y	y	y	y	y	y	y	y	
Continuity (how long are the historical series for the indicator available?)	y/n	y	y	y	y	y	n	n	n	n	
Cost effectiveness	y/n	y	y	y	y	y	y/n	y/n	y/n	y/n	
Theoretical validity (how well the indicator represents the subject of interest.	y	y/n	y	y/n	y/n	y	y	y	y	y	
Reliability (depending on the source: how good and valid is the figure given by the indicator)	n	y	y	y	y	y	y	y	y	y	
Interpretability	y	y/n	y	y	y	y	y	y	y/n	y	
Coverage (is the indicator available for all countries?)	y/n	y	y	y	y	y	y/n	y/n	y/n	y/n	
Final score	10.5	12.5	16	14.5	15	16	11.5	11.5	n. r.^§^	12.5	

*PYLL: Potential years of life lost; °DALY: Disability adjusted life years; §n.r.: not of public health relevance

**Table 3:** The final set of indicators and results of compatibility with EU legislation

	**Topic of the indicator**	**Compatibility with EU legislation**
**State**	Age of vehicle fleet	Compatible
**Exposure**	Time spent on the road and distance travelled	Compatible
**Risk factors**	% of cars exceeding speed limits	compatible (focus point of EU actions)
	Mortality due to drunk driving	compatible (focus point of EU actions)
	Seat belt use, child restraints, helmets	Compatible
**Event**	Road accident rate	compulsory only if generates injury, not harmonised
**Effect**	Death rates	compulsory for MS, harmonised
	***Years of life lost***	Compatible, computed using mortality
	Injury	compulsory for MS, but not harmonised
	***Disability***	Compatible, computed using mortality and morbidity

**Table 4:** Results of feasibility study

**DPSEEA**	**Indicator**	Availability*	Quality*	Comparability*	Policy Relevance*	**Overall Readiness§**	**Feasibility**	**Comment**
State	Age of vehicle fleet	2,00	2,00	2,00	1,60	a	Ready and recommended for immediate implementation	It is reported and discussed on the EEA reports [16]
Exposure	Person time spent on the road	1,14	1,25	1,17	0,83	A to d	Desirable, though requiring further developmental work	EUROSTAT reports the results of the "Time Use Surveys" for the years 1998–2002 in ten European Countries, with some estimates of time spent on the road[15]. EUROSTAT proposed this indicator as voluntary collection.
Exposure	Passengers-kilometres by mode of transport	1,71	1,43	1,43	1,57	a	Ready and recommended for immediate implementation	It is ready, figures are currently present in the international databases. The only limitation is that this indicator does not collect information on distances travelled by human-powered modes of transport.
Risk factor	Use of vehicle safety device	1,14	1,25	1,17	0,83	A to d	Desirable, though requiring further developmental work	
Risk Factor	Mortality due to drunk driving	1,71	1,57	1,29	1,14	A to d	Desirable, though requiring further developmental work	It is both a primary and a secondary risk factor of traffic accident morbidity and mortality.
Risk factor	Speed limit excesses	1,60	1,50	1,25	1,20	A to d	Desirable, though requiring further developmental work	There was no international database identified for this indicator. Therefore, it was agreed that this indicator will be monitored as a pilot indicator on a voluntary basis. In the meantime, according to the relevance of this indicator with respect to the prevention it was recommended that this indicator be proposed to the Eurostat survey to develop more standardized assessment methods.
Effect	Mortality due to road accidents	2,00	2,00	1,83	1,50	a	Ready and recommended for immediate implementation	
Effect	Injury rate	1,71	1,43	1,57	1,29	a	Ready and recommended for immediate implementation	Readily available from CARE-Community Road Accident Database OECD/IRTAD. Improvements in quality and comparability are to be recommended.
Effect	Potential Years of Life Lost	1,60	1,60	1,60	1,50	A to c	Ready, but not feasible for immediate implementation	It is directly calculated from mortality figures. The life expectancy at every age is easily available by the demographic statistics.
Effect	DALY lost for road accidents	1,75	1,25	1,50	1,20	A to d	Desirable, though requiring further developmental work	It is calculated from mortality and injury. To calculate DALYs, disability weights for different countries are necessary. The World Bank has proposed an algorithm to calculate DALYs [14].
Event	Road accident rate	2,00	1,67	1,67	1,80	a	Ready and recommended for immediate implementation	The road accident rate is collected by almost all the European MS and is available in the CARE (Community Road Accident Database)[7]

* Result of feasibility: the figures indicate the mean of the single National Focal Point evaluation scores, the range of the score was from 0 (poor) to 2 (good).

§ Range of the readiness score among countries, the codes are as follows: a) immediately; b) by the end of 2004; c) by the end of 2005; d) after 2006
