# Supplementary material for: Defining a common set of indicators to monitor road accidents in the European Union
Source: BMC Public Health. 2006 Jul 11;6:183. doi: 10.1186/1471-2458-6-183 (PMC1539004; doi:10.1186/1471-2458-6-183)
Supplement: Additional File 1 — DPSEEA model and selected indicators. Application of the DPSEEA model to the road traffic accident field. [file 1471-2458-6-183-S1.doc]

Appendix. List of indicators in the EU-15 Member States. 2002.
